# Management strategies for lower urinary tract symptoms (LUTS) among people with multiple sclerosis (MS): a qualitative study of the perspectives of people with MS and healthcare professionals

**DOI:** 10.12688/hrbopenres.12960.1

**Published:** 2019-11-18

**Authors:** Hawra B. Al Dandan, Rose Galvin, Doreen McClurg, Susan Coote

**Affiliations:** 1 School of Allied Health, Faculty of Education and Health Sciences, University of Limerick, Limerick, Ireland; 2 Imam Abdulrahman Bin Faisal University, Dammam, Saudi Arabia; 3 Health Research Institute, University of Limerick, Limerick, Ireland; 4 Aging Research Centre, University of Limerick, Limerick, Ireland; 5 Nursing, Midwifery and Allied Health Professions Research Unit, Glasgow Caledonian University, Glasgow, UK

**Keywords:** Multiple sclerosis, Neurogenic bladder, management strategies, qualitative study

## Abstract

**Background:** Neurogenic lower urinary tract dysfunction (NLUTD) is defined as a lower urinary tract dysfunction secondary to confirmed pathology of the nervous system. NLUTD is common in people with multiple sclerosis (MS), with prevalence estimates ranging from 49% to 92%. Managing NLUTD is complex and can be comprised of pharmacological and non-pharmacological interventions. Qualitative research exploring perspectives of people with MS and healthcare professionals on living with and managing NLUTD symptoms is sparse. This study aims to explore the perspectives of people with MS and healthcare professionals on managing NLUTD symptoms.

**Methods:** A qualitative descriptive approach will be applied in this study using audio-recorded semi structured interviews for people with MS and healthcare professionals. The Consolidated Criteria for Reporting Qualitative Studies (COREQ) guidelines will be used to standardize the conduct and reporting of the research. People with MS will be recruited through a gatekeeper at MS Ireland. Healthcare professionals will be recruited through gatekeepers at Irish Practice Nurses Association, Continence Foundation of Ireland, Irish Society of Chartered Physiotherapists, and Physiotherapists Interested in MS Group. Interviews will be transcribed and exported to NVivo software package (Version 12) for analysis. Data will be collectively synthesised using thematic analysis.

**Conclusion: **It is anticipated that exploring perspectives of people with MS and healthcare professionals on managing symptoms (including current practice) of NLUTD in MS will assist in the development of an evidence-based and stakeholder informed intervention for NLUTD in people with MS.

## Introduction

Neurogenic lower urinary tract dysfunction (NLUTD) is defined as lower urinary tract symptoms secondary to neurological disease or central nervous system (CNS) injury ^1– 4^ that results in disturbance of ascending or descending pathways to the bladder ^5^. The International Continence Society (ICS) classify NLUTD based on clinical symptoms including storage phase symptoms, voiding phase symptoms, and post-micturition symptoms ^1^. A systematic review of literature demonstrates that lower urinary tract dysfunction is prevalent among people with MS with a pooled prevalence of 68.41% using self-report outcome measures and 62.18% when using the objective measure of urodynamics ^6^. Storage phase symptoms such as frequency, urgency, and/or nocturia appear to be the most prevalent symptoms among people with MS. However, both storage and voiding complaints have been estimated at 50% among people with MS ^7, 8^.

NLUTD among people with MS is associated with significant morbidity that results in activity limitations and reduced health-related quality of life ^9– 13^. Previous research has demonstrated that the presence of bladder related symptoms is associated with worse physical functioning among people with MS regardless of disease duration ^9, 10^. Previous studies also showed a significant correlation between urinary symptoms and a negative impact on emotional health, ability to perform household chores, and physical recreation ^11, 13^. In addition, urinary incontinence has been identified as a factor associated with increased risk of falling among people with MS aged from 45 – 90 years old ^14^.

Management of NULTD is complex and can be comprised of pharmacological and non-pharmacological interventions. In this study we are interested in focusing on non-pharmacological treatments. There is evidence to support the use of pelvic floor muscle exercises, electrotherapy and education as management strategies for neurogenic bladder ^15– 24^. A recent review found some promising evidence to support the use of a new, non-invasive tibial nerve stimulation as an option for NLUTD among people with MS ^25^. Qualitative studies involving perspectives of healthcare professionals and patients on therapeutic interventions play an essential role in incorporating practice-based evidence in to evidence-based practice to improve health outcomes ^26, 27^. A thorough understanding of people with MS and healthcare professionals’ views of various treatment options for urinary symptoms among people with MS will assist in the development of an intervention that aims to improve management of urinary symptoms among people with MS. 

Despite the high prevalence of NLUTD among people with MS, there are a lack of qualitative studies exploring the views of people with MS and healthcare professionals on the management of LUTS and their views on using transcutaneous tibial nerve stimulation (TTNS) as an option to reduce urinary symptoms. A review of qualitative studies relating to NLUTD in MS identified three older studies focused on self-management strategies of urinary incontinence among women ^28, 29^ and men ^30^ with MS. The studies demonstrated the strategies undertaken by patients to control their symptoms of incontinence with no detailed information provided for all types of LUTD. A further qualitative study explored how bladder dysfunction interferes with quality of life in MS ^12^. In terms of healthcare professionals, two qualitative studies were identified. One study explored healthcare professionals beliefs toward bladder dysfunction in MS and how beliefs affect their practice and knowledge regarding bladder dysfunction ^31^, while the second study showed that healthcare professionals require clear evidence-based guidelines focusing on catheter design, appropriate training on the use of single and multi-use catheter to facilitate patient selection of the catheter that is most applicable to their needs ^32^. Therefore, to date no qualitative studies have been identified that focuses on the experiences and current practices of healthcare professionals including TTNS in managing NLUTD in MS. The aims of this study are to: 1) explore the experiences of people with MS in managing NLUTD; 2) explore the views of people with MS on TTNS in managing their NLUTD; 3) explore the healthcare professionals’ role (current practice) in managing NLUTD among people with MS; 4) explore healthcare professionals’ views on TTNS as an option to manage NLUTD in MS.

## Methods

The conduct and reporting of this study is in accordance with Consolidated criteria for reporting qualitative research (COREQ) ^33^. Ethical approval has been granted by the Faculty of Education and Health Sciences Research Ethics Committee at the University of Limerick [Ref 2019_05_18_EHS].

### Research team roles

All interviews will be conducted, transcribed, and analysed by Hawra Al Dandan (HD), a physiotherapist and a PhD candidate at the University of Limerick. HD has completed a qualitative research methodology module that focused on data analysis and she completed an advanced training workshop for Nvivo software. HD contributed to the conceptualization of this research, and will contribute to data curation, data analysis, writing both in the original draft preparation, and in the editing and review of manuscript. Dr Rose Galvin (RG) is a senior lecturer in physiotherapy and experienced researcher in quantitative and qualitative research. RG will provide critical feedback on the transcribed interviews, data analysis, and writing of the manuscript. Prof. Susan Coote (SC) is an associate professor in physiotherapy and experienced researcher in quantitative and qualitative research methods in people with multiple sclerosis. SC has contributed to the conceptualisation of this study and will play a role in providing feedback of the data analysis stage and editing and review of the manuscript. Prof. Doreen McClurg (DM) is a professor of physiotherapy and experienced quantitative and qualitative researcher in bladder and bowel dysfunction among people with MS. DM has contributed to the conceptualisation of this study and will provide feedback on the data analysis, editing and review of the manuscript, and will contribute to the dissemination stage.

### Study design

A qualitative descriptive (QD) approach was chosen for this study. This approach explores general beliefs and views of naturalistic inquiry that expose the experiences described by target populations. It is a common method to gain an insight into a specific topic from the perspectives of participants ^34– 36^. QD has been recommended for use in health care contexts among healthcare professionals and patients because it provides first insight into patient’s and clinicians’ views and experiences within a specific topic ^36^.

### Sampling

A mixed purposive sampling technique will be used in this study ^37^. Purposive sampling allows the selection of information-rich cases for the purposes of a specific study, which results in an in-depth understanding rather than generalisations ^34, 37– 40^. For people with MS, criterion purposive sampling will be used based on predefined inclusion/exclusion criteria ^37^. A representative sample of participants will be sought to participate based on sex, type of MS, disability level, and duration of urinary symptoms.

For healthcare professionals, snow-ball purposive sampling will be used ^37^. It is a non-random sampling technique known as referral sampling where existing research participants recruit a further participant who is information-rich in the field from their acquaintances, and so on ^41^ to ensure continuation of sampling through social networking ^40, 42, 43^.

### Sample size

Sample size will be informed by data saturation ^44^. It is anticipated that 10–15 interviews will be conducted with people with MS, and 5–10 interviews will be conducted with healthcare professionals.

### Recruitment of research participants

For people with MS, recruitment letters and the study information sheet with contact details for the study investigators will be sent through a gatekeeper at MS Ireland ^45^. People with MS will be included if they meet the following inclusion criteria: >=18 years old; have at least one bladder related symptom. People with MS will be excluded if they are unwilling or unable to give informed consent; have an indwelling urethral catheter or indwelling suprapubic catheter; or are pregnant during the data collection phase.

For healthcare professionals, emails will be distributed to healthcare professionals through gatekeepers at Irish Practice Nurses Association, Continence Foundation of Ireland, Irish Society of Chartered Physiotherapists, and the Physiotherapists Interested in MS Group. In Ireland, these are the core healthcare professionals who interact with individuals with MS who may have urinary symptoms. The study information sheet and contact details for the study investigators will be attached in the body of the email ^45^. Healthcare professionals will be eligible if they are treating or have treated bladder related symptoms among people with MS. Completion of the written informed consent form ^45^ will be a prerequisite for study participants as articulated in the Health Research Regulations 2018.

### Data collection

Telephone interview has been shown to be an effective method for collection of qualitative data, this includes data related to sensitive topics due to the following benefits: obtaining rich data; more flexibility for scheduling; convenience for participants including clinicians; enhanced access to geographically dispersed areas; less time-consuming for the researcher and participant in terms of travel; and more cost-effective ^46, 47^. Audio recording, semi-structured interviews and written notes will be undertaken for the purposes of this study ^38, 40, 48– 50^. One interview will be conducted for each participant by HD, with no prior established relationship with participants. The interviews will be conducted in a private room in the School of Allied Health at the University of Limerick. An independent researcher will present during the interview as a note-taker. Each interview is expected to last approximately 30 minutes for people with MS and up to 60 minutes for healthcare professionals.

### Interview guide

The interview questions were developed by the researchers by reviewing existing literature relating to patient and carer experiences of incontinence among MS ^51^. The interview questions were based on the principles of constructing semi-structured interviews reported in the literature ^38^. Open-ended interview questions were chosen as they allow the participants to explore and discuss their experiences in managing bladder related symptoms. The interview guide is available as *extended data* of this manuscript ^45^.

Data will be handled confidentially and will be stored in accordance with Data Protection Policy at the University of Limerick and in line with the Health Research Regulations 2018. All recordings will be stored anonymously, securely and confidentially in the principal investigator office in the School of Allied Health at the University of Limerick. RG the Principal Investigator and the co-investigators HD, SC and DM will have access to the data collected. At the end of each interview, HD will transfer the audio recordings to a password-protected laptop and anonymised transcripts will be saved in the same laptop. Upon completion of transcription of interviews, pseudonyms will be placed instead of the participants’ actual names. Audio recordings will be deleted once transcription has been completed. The data will be stored for seven years then all hard copies will be shredded, and electronic files will be deleted permanently. Prior to the analysis stage, participants will be offered the opportunity to review the transcript document for comments, and corrections to ensure accuracy of the interview transcripts ^40^.

### Data analysis

In this study, NVivo software package (version 12) will be used to import transcripts, organize, store, and retrieve data to be ready for analysis. Data will be collectively synthesized using a reflexive thematic analysis, inductive approach ^52– 54^. In this approach, the coding process is data driven rather than by researchers’ pre-existing theories or researcher’s analytical prejudices. This type of analysis provides six systematic phases, which will be conducted by HD with critical review and feedback from RG. Phase 1 includes familiarization with collected data, while phase 2 includes an in-depth engagement with data and extraction of initial codes by highlighting the actual words from participants. Phases 3 and 4 involve generating themes by sorting different codes to form potential themes and then reviewing the link between the themes and the original dataset. These phases also involve refining themes to code any additional data. In phases 5 and 6, themes will be defined collectively by giving a name to each theme through review and consultation between study investigators. Finally, a narrative report will be provided by HD and reviewed by RG to ensure a clear audit trail of methodology during the research process.

According to the recent literature ^54^, the approach to reflexive thematic analysis highlights the active role of the researchers as the primary tool to ensure a good quality study. The approach sees coding as a flexible, organic and subjective process that aims to reflect how the researchers are conceptualizing the data. To this end, the coding process captures the relationship of the data to the research questions and serves to generate distinctive themes that capture patterns of shared meaning across the dataset, underpinned by a central organizing concept and generating an interpretative rather than descriptive analytic report. In the current study, methodological rigour will be addressed by: adhering to a 15-point checklist of criteria for good thematic analysis ^55^; the use of a reflective report, which is considered an essential step in qualitative research to enhance transparency and trustworthiness; sharing the coding process with the study research team; and by using field notes to document initial impressions during data collection ^56, 57^.

## Dissemination

Subsequent to the analysis stage, key themes and a summary of findings will be shared with participants. While this qualitative study will inform the intervention of a future pilot study for neurogenic bladder among people with MS, the results will also be submitted for publication. Abstracts will be submitted to relevant national and international conferences. Locally, the study results will be shared with healthcare professionals who participated in the study and with the Physiotherapists Interested in MS Group. For people with MS, the results will be submitted to MS Ireland newsletters through a gatekeeper at MS Ireland.

## Study status

The study is due to commence in November 2019.

## Conclusion

To the authors’ knowledge, no studies have been conducted to explore the experiences and perspectives of healthcare professionals and people with MS in management strategies of NLUTD in MS. It is anticipated that understanding and exploring management strategies and current practice of NLUTD among people with MS will assist in the development of an intervention that may serve to improve management of urinary symptoms among people with MS.

## Data availability

### Underlying data

No underlying data is associated with this article.

### Extended data

Open Science Framework: Management strategies for lower urinary tract symptoms (LUTS) among people with multiple sclerosis (MS): a qualitative study of the perspectives of people with MS and healthcare professionals. https://doi.org/10.17605/OSF.IO/64ETG ^45^

This project contains the following extended data:

- Appendices.docx (recruitment letters, participant information sheets, consent forms and demographic data sheets for people with MS and healthcare professionals)
- Interview Guide.docx (interview guide for people with MS and healthcare professionals)

Data are available under the terms of the Creative Commons Zero “No rights reserved” data waiver (CC0 1.0 Public domain dedication).
