# Supplementary material for: Management strategies for lower urinary tract symptoms (LUTS) among people with multiple sclerosis (MS): a qualitative study of the perspectives of people with MS and healthcare professionals
Source: HRB Open Res. 2019 Nov 18;2:31. [Version 1] doi: 10.12688/hrbopenres.12960.1 (PMC7140768; doi:10.12688/hrbopenres.12960.1)
Supplement: Peer review history

# Peer review: Reviewer response for version 1

**DOI:** 10.21956/hrbopenres.14043.r27231

**Published:** 2020-03-25

**Authors:** Christine W. Bradway, Nicole Zhang

**Affiliations:** 1 Hartwick College, Oneonta, NY, USA; 2 School of Nursing, University of Pennsylvania, Philadelphia, PA, USA

Overall, the study addressed a paucity in the literature. Surely, more qualitative data regarding lower urinary tract symptoms (LUTS) in clients with multiple sclerosis is an important area of research. As the authors allude to, this information would be useful in designing programs for clients with these concerns.

 The manuscript clearly describes the aims of the study and methodology. Generally, the aims of the study can be addressed using this methodology. Two out of four of the study aims focused on transcutaneous tibial nerve stimulation (TTNS). However, the information in the background/literature review did not address TTNS. As such, how do the authors support selections of those aims? Adding more information to the background/review of the literature would be helpful to support the selection of these aims. Alternatively, the authors could also explain why selecting those two aims for the exploratory qualitative designed study is important.

 Overall, this is a well written article describing the study protocol and we would recommend approval with attention to the suggested minor changes described above.

We confirm that we have read this submission and believe that we have an appropriate level of expertise to confirm that it is of an acceptable scientific standard.

# Peer review: Reviewer response for version 1

**DOI:** 10.21956/hrbopenres.14043.r27128

**Published:** 2020-03-11

**Authors:** Apostolos Apostolidis

**Affiliations:** 1 Aristotle University, Thessaloniki, Greece; 2 Papageorgiou General Hospital, Thessaloniki, Greece

This is definitely an original research topic, involving both patients and physicians, who are commonly not properly communicating neither upon studies design nor when applying treatment for LUTS. The qualitative study design is analytically presented, and admittedly, all the design until data collection and handling appears to be impeccable.

 However, I could not find the interview tool, thus I could not make an assessment of this, which is why I answered 'Partly’ to the question ‘ *Are sufficient details of the methods provided to allow replication by others?* Also, a query would be why the methodology of this research needs to be published separately, particularly since already approved by the local University and registered online.

I confirm that I have read this submission and believe that I have an appropriate level of expertise to confirm that it is of an acceptable scientific standard.

# Response

**Published:** 2020-03-23

**Authors:** Hawra Al dandan

**Affiliations:** university of limerick, Ireland

Dear Dr Apostolos Apostolidis, 

Many thanks for your feedback related to the protocol and my sincere apology for such delay in my response. As you know the previous week was so difficult as a result of such global circumstances. 

Regarding the interview tool, it could be reviewed online following the link provided in the protocol. 

Thank you
